# 17. CANNABIDIOL AS A TREATMENT IN DIFFERENT STAGES OF PSYCHOSIS- EFFICACY AND MECHANISMS

**DOI:** 10.1093/schbul/sby014.064

**Published:** 2018-04-01

**Authors:** Sagnik Bhattacharyya

**Affiliations:** Institute of Psychiatry, King’s College London

## Abstract

**Overall Abstract:** While drugs that target the dopaminergic and glutamatergic neurotransmitter systems have been extensively investigated as treatments for psychosis, there has been increasing attention in recent years on the endocannabinoid system as a therapeutic target. The CB1 receptor, the main central cannabinoid receptor is ubiquitous and modulates the function of several neurotransmitters, including dopamine and glutamate. A growing body of evidence suggests that psychosis is associated with alterations in the endocannabinoid system, independent of exposure to cannabis.

The CB1 receptor is the main molecular target for delta-9-tetrahydrocannabinol (THC), the major psychoactive ingredient in cannabis. THC is responsible for the psychotogenic effects of cannabis, and is a partial agonist at the CB1 receptor. On the other hand, Cannabidiol (CBD), the second major constituent of cannabis is a non-psychoactive compound that may have an inverse agonist/antagonist effect at CB1 receptors, in addition to a range of other possible mechanisms of action. Interest in the therapeutic potential of CBD stemmed from evidence that it has broadly opposite effects to that of THC, at both the neural and the behavioural level in healthy individuals. Consistent with these results independent evidence that CBD has antipsychotic and anxiolytic properties in patients with mental health disorders has been accumulating. The absence of significant adverse effects associated with CBD, is a critical advantage in relation to the treatment of patients in the various stages of psychosis. Given its tolerability profile, CBD is a treatment of particular interest not just in those with chronic psychosis as in schizophrenia, but also in those in the earlier stages of psychosis. However, published evidence regarding the therapeutic efficacy of CBD as a treatment in psychosis has been limited except a small randomized clinical trial (RCT) some years ago (Leweke et al 2012). Furthermore, the mechanisms that may underlie the beneficial effects of CBD are unclear.

This symposium will bring together state of the art evidence regarding the efficacy of CBD in the different stages of psychosis – from those at clinical high-risk (CHR), through early psychosis to chronic schizophrenia. Furthermore, data from animal and human studies will be presented to give an understanding of the potential mechanisms that may underlie the therapeutic effects of CBD.

The first speaker (Prof Crippa) will set the scene by presenting evidence regarding the different potential mechanisms of action that may underlie the antipsychotic effect of CBD.

The second speaker (Prof McGuire) will present the results of a 6-week placebo-controlled RCT demonstrating the efficacy of CBD as an add-on to existing antipsychotic treatment in schizophrenia.

The third speaker (Dr. Ranganathan) will present the results from an ongoing, placebo-controlled RCT using an within-subject, crossover design to show the effects of 4-week CBD treatment on psychotic symptoms, cognition and electrophysiological markers in patients with established psychosis.

Finally, the fourth speaker (Dr. Bhattacharyya) will present the results from a recently completed proof-of-concept study demonstrating the efficacy of short-term CBD treatment on symptoms and distress in CHR patients as well as on neurocognitive substrates implicated in the CHR state.

Evidence presented here will be discussed by Prof D’Souza, who is an internationally recognized expert in cannabinoid pharmacology and experimental therapeutics development.

